# Invertase-nanogold clusters decorated plant membranes for fluorescence-based sucrose sensor

**DOI:** 10.1186/s12951-015-0089-1

**Published:** 2015-04-12

**Authors:** Dipali Bagal-Kestwal, Rakesh Mohan Kestwal, Been-Huang Chiang

**Affiliations:** Institute of Food Science and Technology, National Taiwan University, No.1, Roosevelt Road, section 4, Taipei, Taiwan

**Keywords:** Nanogold clusters, Gold nanoparticles, Invertase, Onion membrane, Sucrose, Glucose, Analyte, Fluorescence, Quenching-based biosensor

## Abstract

In the present study, invertase-mediated nanogold clusters were synthesized on onion membranes, and their application for sucrose biosensor fabrication was investigated. Transmission electron microscopy revealed free nanoparticles of various sizes (diameter ~5 to 50 nm) along with clusters of nanogold (~95 to 200 nm) on the surface of inner epidermal membranes of onions (*Allium cepa L.*). Most of the polydispersed nanoparticles were spherical, although some were square shaped, triangular, hexagonal or rod-shaped. Ultraviolet–visible spectrophotometric observations showed the characteristic peak for nanoparticles decorated invertase-onion membrane at approximately 301 nm. When excited at 320 nm in the presence of sucrose, the membranes exhibited a photoemission peak at 348 nm. The fluorescence lifetime of this nanogold modified onion membrane was 6.20 ns, compared to 2.47 ns for invertase-onion membrane without nanogold. Therefore, a sucrose detection scheme comprised of an invertase/nanogold decorated onion membrane was successfully developed. This fluorescent nanogold-embedded onion membrane drop-test sensor exhibited wide acidic to neutral working pH range (4.0-7.0) with a response time 30 seconds (<1 min). The fabricated quenching-based probe had a low detection limit (2x10^−9^ M) with a linear dynamic range of 2.25x10^−9^ to 4.25x10^−8^ M for sensing sucrose. A microplate designed with an enzyme-nanomaterial-based sensor platform exhibited a high compliance, with acceptable percentage error for the detection of sucrose in green tea samples in comparison to a traditional method. With some further, modifications, this fabricated enzyme-nanogold onion membrane sensor probe could be used to estimate glucose concentrations for a variety of analytical samples.

**Electronic supplementary material:**

The online version of this article (doi:10.1186/s12951-015-0089-1) contains supplementary material, which is available to authorized users.

**Graphical abstract:**
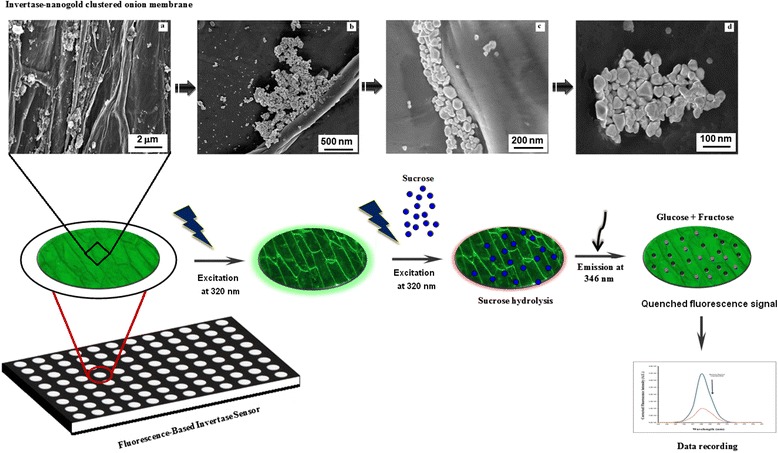
Synthesis and characterization of invertase assisted nanogold clusters on onion membranes and their application for fluorescence-based sucrose sensor.

## Introduction

Metal nanoparticles are outstanding building blocks for fabrication of biosensors, due to their surface plasmon resonance shifts in response to a biorecognition event. Properties of nanoparticles vary in accordance with their size and composition, which facilitates diverse applications in various areas including catalysis, sensors and medicine. Production of nanoparticles can be achieved through chemical, physical or biological methods. Among them, there has been considerable attention focused on biological methods for synthesis of metallic nanoparticles, as there is a vast array of biological resources available in nature [1,2]. Furthermore, the biological approach to nanoparticles synthesis is also a low cost, non-toxic, biocompatible and environmentally friendly process.

Enzymes can work as nanoreactors that allow generation of nanostructures often of controlled size by limiting the rate of nucleation of nascent nanocrystals [3]. For example, enzyme-guided nanoparticles have been used for fabrication of biosensors that can detect prostate-specific antigen (a biomarker of prostate cancer), with outstanding sensitivity. The development of optical sensors using enzyme-stimulated synthesis of metallic nanoparticles has been also reported by Willner et al. [4]. According to their study, in the presence of bovine serum albumin, bacterial protease not only mediated biosynthesis of gold (Au) nanoparticles, but also acted as reducing and shape-directing agents. *In vitro* enzymatic synthesis of Au nanoparticles using alpha-NADPH-dependent sulfite reductase and phytochelatin was reported by Kumar et al. [5]*.* Kalishwaralal et al., investigated biosynthesis of gold, silver, and gold-silver alloy nanoparticles by harnessing free and exposed thiol groups of α-amylase [1]. Furthermore, the native reducing properties of plant proteins have also been harnessed for synthesis of Au nanoclusters [6,7]. Mittal et al., has reviewed the methods for making nanoparticles using plant extracts and the potential applications of these nanoparticles for various applications [8]. Moreover, the biosynthesis of Au nanoparticles by many plants such as *Medicago sativa* [9], *Azadirachta indica* [10], *Aloe vera* [11], *Cinnamomum camphora* [12]*, Magnolia kobus*, *Diopyros kaki* [13], *Szyygium aromaticum* [14], *Putranjiva Roxburghii* [15], *Cassia auriculata* [16], among others, has been well documented.

Consumption of sugar added beverages and numerous other foods have increased across the globe. Therefore, quantitative determination of sugar content in food is an important issue. In particular, there is need for a fast, simple, and reproducible method for determination of sucrose content [17]. Fluorescent-sensing systems have become increasingly popular, due to their versatility, ease of use and low cost. Several analytical techniques have been developed, to exploit changes in fluorescence properties of a molecule in different environments, including quenching, Forster resonance energy transfer, and surface-modified fluorescence (FL) [18]. Invertase (INV; β-fructofuranosidase) is an enzyme with a high rate of enzymatic turnover. Under ambient conditions, nanomolar levels of INV are capable of converting a millimolar concentration of sucrose into glucose, making it an ideal catalyst for amplification of ‘turn-on’ signals in sucrose sensors [19]. Consequently, optical nanoprobe utilizing electrospun polyamide meshes containing gold salts and invertase have been reported to be useful for sugar-sensing [20]. Blue and pink colorimetric assays based on sugar and glucose oxidase-assisted synthesis of nanoparticles for sugar detection, have also been reported [17]. However, reports of fluorometric biosensors for sucrose estimation are very few.

The inner epidermal membrane of the onion bulk scales is a good bio-platform to immobilize enzymes, as it has excellent gas and water permeability for substrates and products. Onion membranes (Oms) mainly consist of elongated tubular cells, with blunt or tapered ends, along with numerous guard cells. For biosensors fabrication, this natural membrane is mechanically stronger than other natural membranes, due to its microfibrillar cellulosic elongated tubular structure. Thus, it could be ideal as a biocompatible platform for enzyme immobilization [21,22]. Kumar and Pundir reported immobilization of lipase on onion membrane and its possible commercial application in food-processing industries [23]. A glucose biosensor comprising glucose oxidase/O-(2-hydroxyl) propyl-3-trimethylammonium chitosan chloride nanoparticle-immobilized on the inner membrane of onion and a dissolved oxygen sensor have also been reported [24]. Furthermore, a glucose biosensor based on onion primary cuticula that immobilized glucose oxidase was reported for determining glucose concentrations in human serum [25].

The objective of our study was to synthesis and characterize invertase-nanogold clusters (INV-NAuCs) embedded in plant membranes and investigate their application in designing fluorescent probes for sucrose detection. The novel feature of our proposed method is employment of a new biomaterial along with enzyme for gold nanomaterial synthesis and biosensor development. Various factors that might influence the sensor performance have been investigated. The fabricated drop-test sensor was then used to detect sucrose in various green tea samples to demonstrate its high sensitivity and specificity.

## Results and discussion

### Invertase- mediated nanogold synthesis: UV-Visible studies

**Figure 1:**
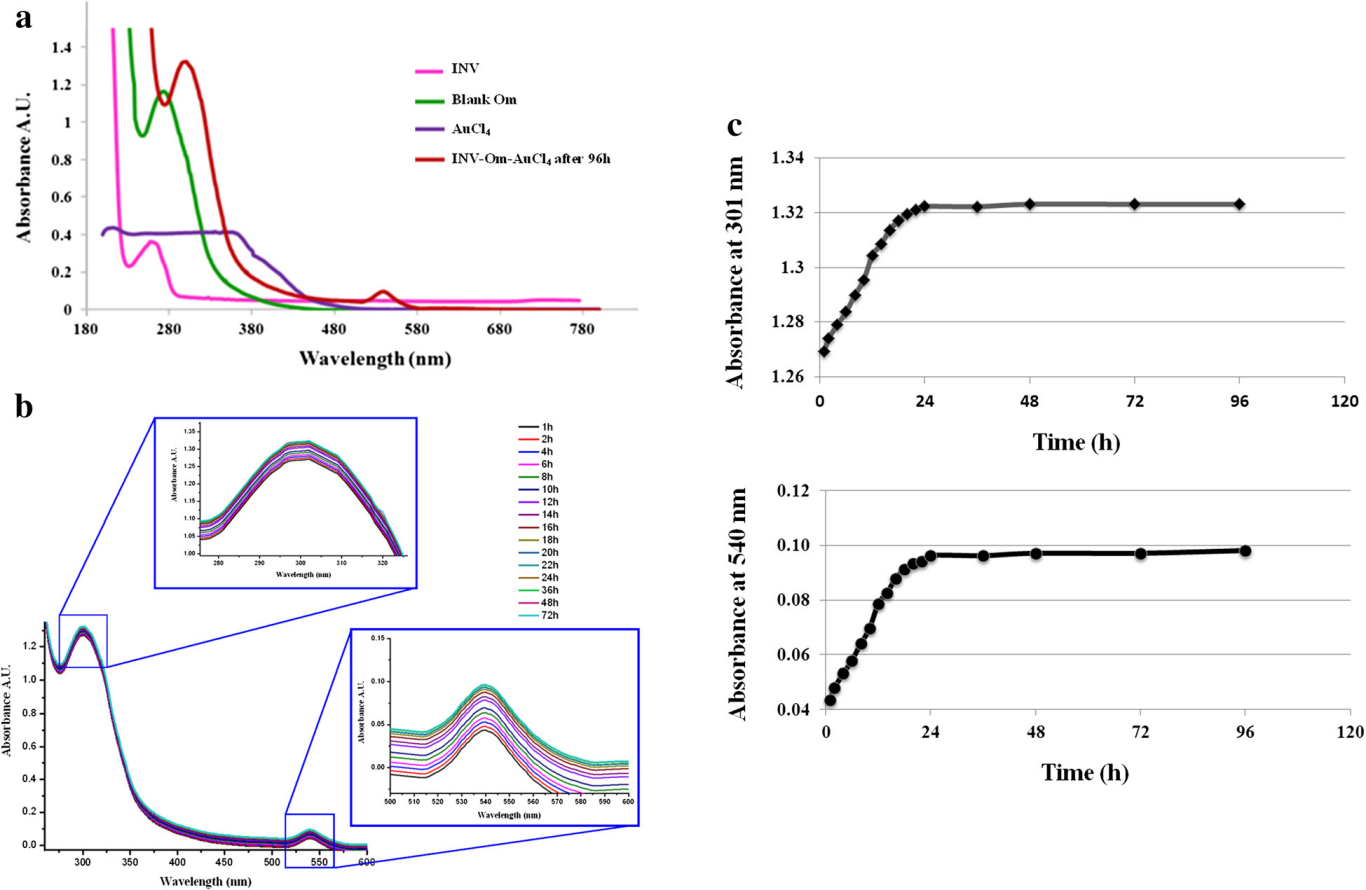
UV-Visible absorption spectra of invertase-onion membranes. **(a)** UV-Visible absorption spectra of invertase, blank onion membrane, AuCl_4_ solution and onion membrane with INV and AuCl_4_ incubated for 96 h in 20 mM acetate buffer solution (pH 5.0). **(b)** Periodical UV-visible absorption spectra of invertase-onion membrane with dispersed nanogold in 20 mM acetate buffer solution (pH 5.0). Inset I: Absorption peak at 301 nm; Inset II: Absorption peak at 540 nm. **(c)** Plot of time versus absorption of nanogold clusters synthesized on an invertase-onion membrane at 301 nm **(a)** and 540 nm **(b)**, respectively.

The UV-Visible absorption spectra of invertase, blank (untreated with invertase) onion membrane, hydrogen tetrachloroaurate (HAuCl_4_) solution and lastly, onion membrane with invertase and HAuCl_4_ for 96 h in acetate buffer (20 mM, pH 5.0) are shown in Figure 1a. The HAuCl_4_ solution had no obvious absorption peak, whereas the spectrum of INV had an absorption peak at 260 nm and blank onion membrane in assay buffer had an absorption peak at 275 nm. After incubating the onion membrane with invertase and gold chloride solution for 96 h, the absorption peak shifted from 275 to 301 nm, which indicated that there was a direct reaction among gold chloride, invertase and onion membrane to form nanogold clusters (NAuCs). Moreover, one minor peak was observed at 540 nm, which is also a characteristic of gold nanoparticles (AuNPs).

Formation of gold nanomaterials was further confirmed by UV–vis spectroscopy. Periodic UV–vis absorption spectra of onion membranes embedded with invertase-nanogold clusters (INV-NAuCs-Om) are shown in Figure 1b. All of spectra displayed the same plasmonic band for major dominant peak I at 301 nm (inset I) and minor surface plasmon peak II at 540 nm (Inset II), which intensified with time. The nanogold biosynthesis process was monitored continuously for 96 h. Absorption intensity increased with duration of incubation, reaching a plateau at 72 h, indicating saturation of nanogold formation on onion membrane after 72 h (Figure 1c). These results are similar to those reported by Parida et al. [6]. Biological activity of INV after nanogold formation was confirmed by DNSA method [26-30].

Nanogold synthesis on the invertase-immobilized onion membrane was consistent with the properties of onion membrane as a reducing and stabilizing agent. A similar approach was used for synthesis of silver nanoparticles, with onion (*Allium cepa*) extract acting as both a reducing as well as capping agent [31]. Parida et al., stated that reduction of gold nanoparticles occurred in onion extract due to the presence of ample vitamin C, citric acid, ascorbic acid, flavonoids and extracellular electron shutters, etc. [6,32]. However, the specific role of the invertase in the synthesis of nanogold has not been well established. One hypothesis is that a high content of vitamin C, flavonoids, thiosulphonates and other organosulfur in onion membranes are directly involved in the gold reduction mechanism. The exposed S–H groups of invertase may allow enzyme binding to the gold ions via gold–S bond without jeopardizing INV structure. The increased rate of NAuC production by the enzyme indicated a rapid reduction of Au^3+^ to Au^0^ by the exposed functional groups of reducing amino acids (*e.g.* the thiol group of cysteine and the tertiary amine group of histamine) [33]. Furthermore, it is possible that intrinsic enzymatic generation of reactive sulfur species develops surface plasmon resonance at gold nanostructures which may turn them into tiny fluorophores. A more in-depth investigate is needed to understand the internal mechanism responsible for the formation of nanoparticles or clusters in the presence of invertase.

### Topological investigation of nanogold membrane

**Figure 2:**
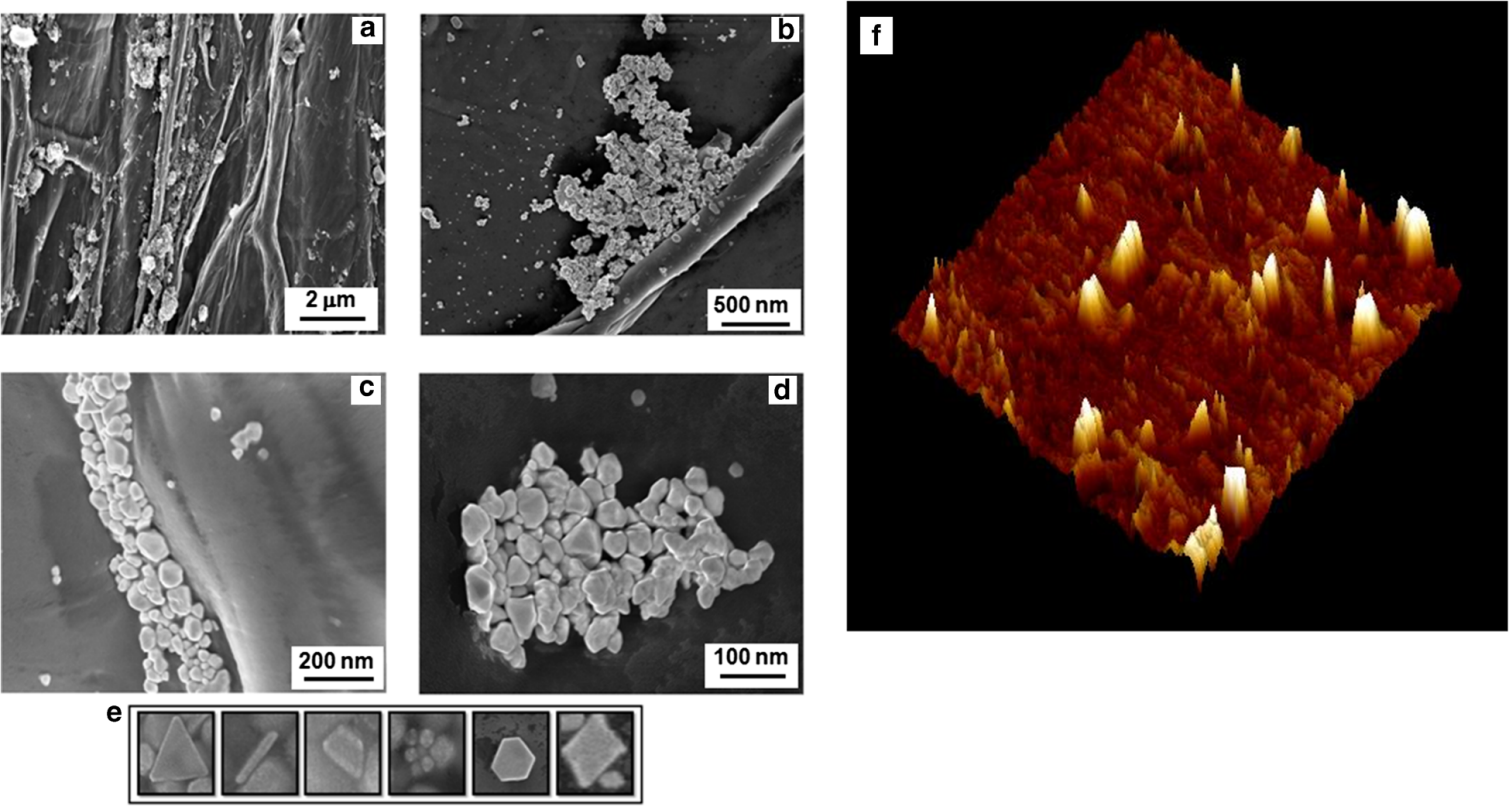
Microscopic images of enzyme-nanogold clusters-onion membrane. SEM images of INV-nanogold onion membrane at various magnifications **(a-d)**; TEM images of various shapes of individual nanoparticles present in nanogold clusters **(e)** and AFM topography of onion membrane showing gold clusters domains **(f)**.

We also analyzed the topography of nanogold using both scanning and transmission electron microscopy (SEM and TEM, respectively). In the SEM images nanoclusters were found aligned with onion epidermal cell walls (Figure 2a-d). The biosynthesized nanogold clusters, ranging in size from 95 to 200 nm, either adhered to or embedded in the membrane. Most of the poly-dispersed nanoparticles were spherical, although other shapes were also visible, e.g*.* square-shaped, triangular, rectangular, hexagonal and cylindrical, but to a lesser extent. Based on transmission electron microscopy (TEM), these nanoparticles were ~5 to 50 nm in size (Figure 2e). Three-dimensional atomic force microscopic (AFM) images of onion membrane also showed prominent domain impressions of gold nanoclusters into the membrane surface of the onion (Figure 2f).

### Characteristic fluorescence spectra of INV- NAuCs- Om

**Figure 3:**
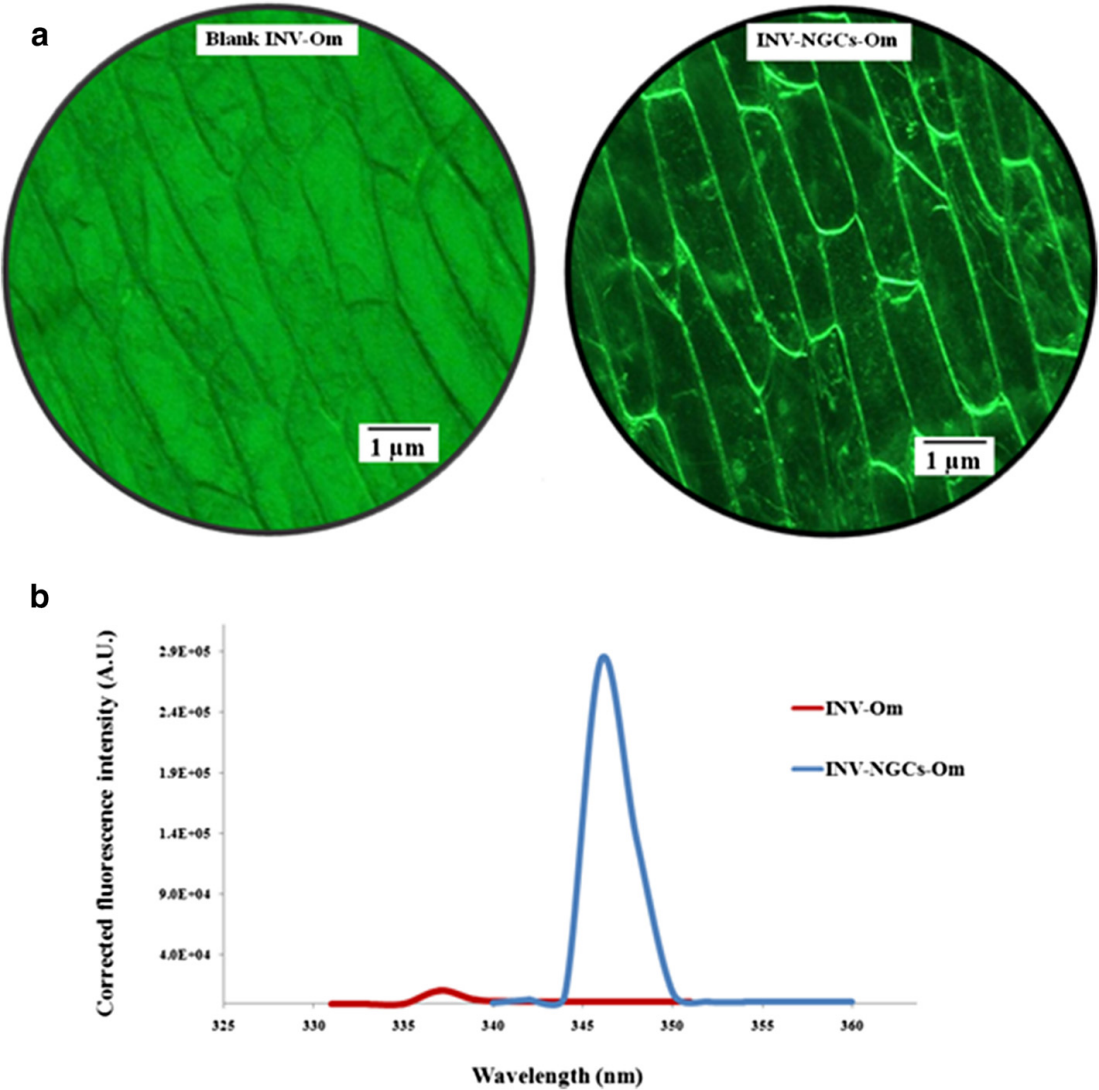
Fluorescence studies of INV-Om and INV-NAuCs-Om. **(a)** Fluorescence images of INV-OM and INV-NAuCs-Om **(b)** Fluorescence spectra of INV-Om and INV-NAuCs-Om at excitation wavelength 320 nm.

Invertase-onion membranes, both with and without nanoparticles, were excited at 320 nm and the spectra were recorded in an emission range of 330 to 700 nm. The image of INV-Om, indicate that the inner epidermal onion membranes with invertase only did not possess fluorescence, whereas onion membrane decorated with gold clusters did have a fluorescent image (Figure 3a). Furthermore, invertase-immobilized onion membrane had a small peak at 337 nm (Figure 3b). Similarly, Hou et al*.,* reported that the free invertase had an emission peak at 335 nm when excited at the same wavelength [34]. The slight red shift in emission peak (~2 nm) may have been due to the immobilization process. The possibility of emissions arising from the reagents (*e.g.* HAuCl_4_, sucrose and the mixture of HAuCl_4_–sucrose), were also examined. Blank onion membranes incubated with reagents showed very weak fluorescence which was similar to that of the background signal. However, INV-NAuCs-Oms in 25 μL acetate buffer (20 mM, pH 5.0) had a photoemission peak at 346 nm. Perhaps the native reducing property of invertase was also harnessed for synthesis, capping and aggregation of gold particles into stable nanogold clusters. The electrostatic bonding and steric protection due to the bulkiness of the protein may also be responsible for stable INV-Om-scaffolds. Similar observations were reported for photoluminescent BSA-protected nanoparticles, with excitation and emission maxima at 320 and 404 nm, respectively [35].

### Fluorescence lifetime and quantum yield of the membrane

**Figure 4:**
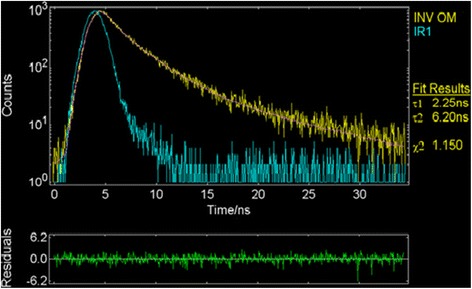
Fluorescence lifetime for INV-NAuCs-Om in aqueous assay buffer solution (yellow line) whereas pink line corresponds to the non-linear least square fit value (*x* ^2^ = 1.150). Blue line represents IR spectrum. The lower panel represents the residual plot of the fit.

The fluorescence lifetime for INV-NAuCs-Om was 6.20 ns and *x*^2^ = 1.150 (Figure 4). In addition, the lifetime for blank Om and INV-Om without nanogold, were 1.23 and 2.47 ns respectively. Therefore, the association of invertase and other proteins with nanoparticles could increase the fluorescence lifetime. The fluorescence average lifetime of INV-nanogold-onion membranes was similar to bovine serum albumin modified gold nanoparticles (BSA-GNPs) previously reported [36].

The quantum yield of gold nanoparticles was conservatively estimated to be 0.065 ± 0.0050 (P = 0.90), approximately eight orders of magnitude greater than that of gold films. Based on the INV-AuNPs-Om high quantum yield (Φ = 0.17 ± 0.004), we may inferred that the membrane is a useful element for fluorescent sensors. In addition, as the invertase-conjugated nano gold particles individually acted as embedded fluorophores in the membranes, it can be concluded that the intrinsic fluorescence of invertase tryptophan was scarcely used. Furthermore, the conservatively estimated quantum yield of INV-gold nanoparticles was found to be approximately seven and twelve orders of magnitude greater than that of blank INV-Om and blank Om, respectively.

### Influence of pH on nanogold clusters synthesis and fluorescence property

The influence of pH on the synthesis of nanogold clusters and their morphological properties was studied and observations are provided in Additional file 1 (S1.1 section). The fluorescence of the INV-NAuCs-onion membranes at various pH levels was also checked. For this purpose, membranes in respective pH solutions were excited in the range of 340 to 360 nm and pH range of 3.0 to 11.0. There were emission peaks at 344, 346 and 348 nm for pH 3.0, 4.0 and 5.0, respectively (Additional file 1: Figure S3), whereas emission peaks for pH 6.0 to 9.0 were all observed at 348 nm. For pH 10.0 and 11.0 the fluorescence peaks were observed at 350 and 353 nm, respectively (Additional file 1: Figure S3 inset). The fluorescence intensity from pH 3.0 to 5.0 increased in a linear fashion, with the maxima at pH 5.0, which could be attributed to the invertase pH optima. Nanogold decorated onion membranes were non-fluorescent when they were excited in the range of 540–600 nm.

### Effect of invertase and HAuCl_4_ concentration on nanogold clusters synthesis

The influence of enzyme concentration on nanogold formation and fluorescence intensity is shown and discussed in Additional file 1: Figure S1.2. The chloroauric acid concentration affected the size of nanogold and assemblies formation. For the concentration of HAuCl_4_ from 0.25 to 1.0 mM, fluorescent gold nanoparticles were formed, with diameters from 2-50 nm (Table 1). As the concentration increased to 1.5 mM, nanoparticles approaching 70 nm (± 32%) in diameter were produced. At 2.0 mM HAuCl_4_, spherical gold particles (diameter ~90 nm) were formed. These gold nanoparticles had surface plasmon ~543 nm without fluorescence properties. However, further increasing the concentration above 2.0 mM had no major effect on particle growth. The surface plasmon band in the gold nanoparticles solution remained close to 543 nm throughout the reaction period. Therefore, we inferred that nanoparticles were dispersed in the aqueous solution, leaving no evidence of aggregation in UV-Vis absorption spectrum. However, at a high gold salt concentration, steric hindrance and salt crowding on the enzyme surface may change protein structure, eventually causing enzyme precipitation with diminished invertase activity. Surface plasmon resonance peaks and fluorescence intensities with respect to gold salt concentration are shown in Table 1. These results were in good agreement with a previous report, in which effects of gold nanoparticle morphology on adsorbed protein structure and function were thoroughly studied [37].

**Table 1:** **Effect of gold concentration on nanogold formation and fluorescence intensity**

**HAuCl**_**4**_ **(mM)**	**Size distribution range (nm)**	**SPR peak I (nm)**	**SPR peak II (nm)**	**Normalized fluorescent intensity (A.U.) (λ**_**ex**_ **= 320 nm, λ**_**em**_ **= 348 nm)**
0.25	2↔10.8	301.3	517	219291
0.50	13.4↔27.2	301.0	538	330387
0.75	15.8↔34.2	301.1	543	302343
1.00	31.1↔47.2	301.3	542	95327
1.25	50↔65.7	301.3	543	53299
1.50	70.4↔81.2	301.5	543	39397
2.00	91.4↔91.2	302.0	544	37159

### Application of the INV-NAuCs-Om sensor for sucrose sensing

Fluorescence measurement allows direct background subtraction strategy while colorimetric assays suffer background interference problem when onion membranes were used directly. Moreover, FL technique is extremely sensitive and fast. FL measurements also provide structure and micro-environment of molecules which help to understand the detailed reaction mechanism. All these special features are important for sensing applications and therefore fluorescent sensors are more attractive as compare to colorimetric sensor.

**Figure 5:**
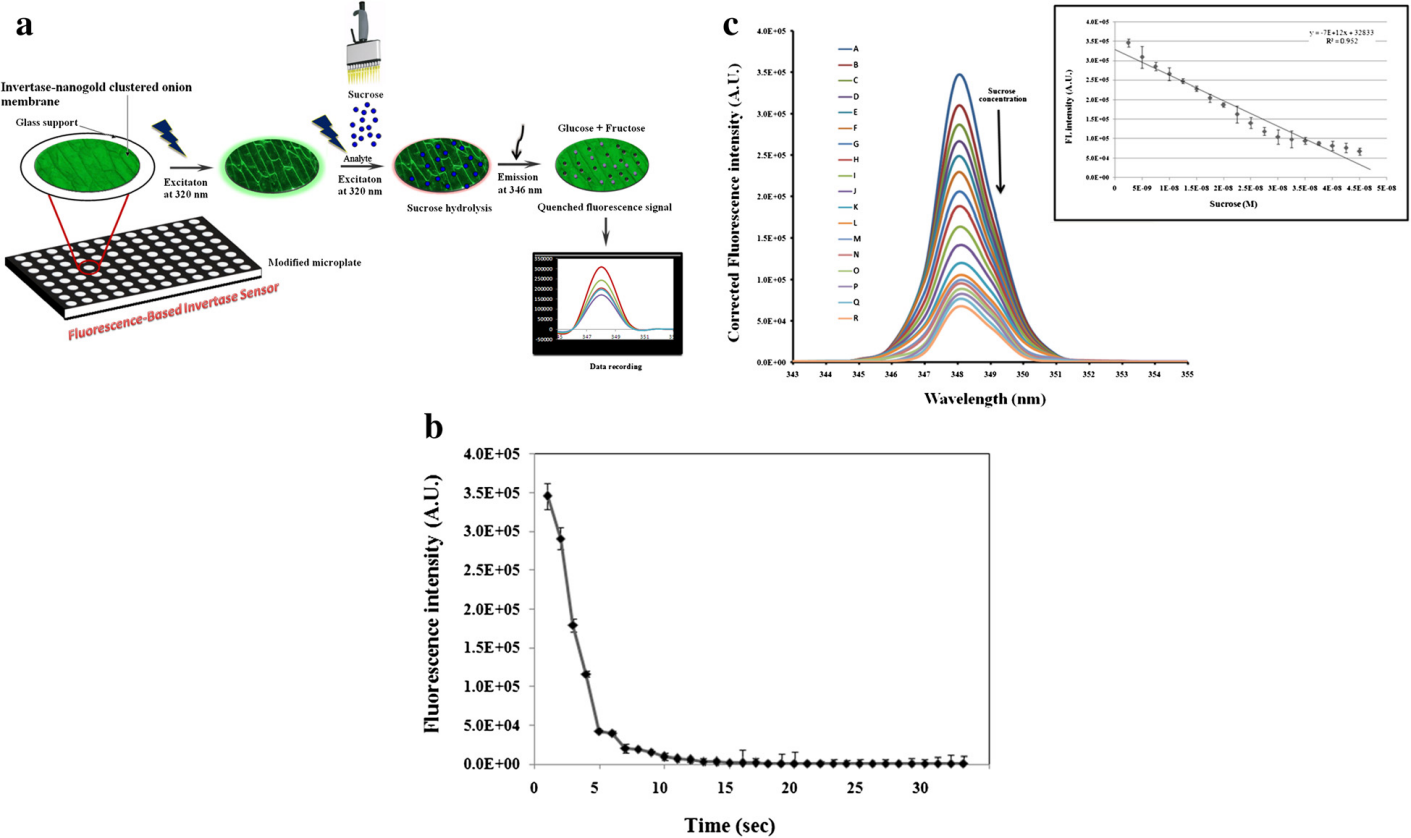
Fluorescent properties of INV-NAuCs-Om. **(a)** Schematic representation of a fluorescent sucrose-sensing mechanism using nanoparticles decorated invertase-onion membrane modified microplate sensor. **(b)** Quenching signal response of sucrose on INV-NAuCs-Om. **(c)** Fluorescence spectra of Au-Particles-INV-onion membrane in presence of various concentrations of sucrose (0.2x10^−8^ to 4.25x10^−8^ M). Inset I: FL intensity as function of Sucrose concentration. Inset II: Calibration plot using ΔFL *vs*. sucrose concentration (0.2x10^−8^ to 4.25x10^−8^ M).

A schematic representation of the sucrose sensing mechanism behind a microplate sensor modified with a nanoparticle-decorated invertase-onion membrane is shown in Figure 5a. The sucrose-sensing performance of INV-NAuCs-Om was evaluated with fluorometric measurements, which showed a slight blue shift (emission at 348 nm) after sucrose addition at excitation wavelength 320 nm. Furthermore, sucrose was a strong quencher for INV-NAuCs in the UV region. A similar quenching effect was reported by many researchers measuring monosaccharides; therefore, this property is exploited for analyte sensing [38,39]. Consequently, this enzyme-based onion membrane assembly was used as a fluorescence-based optical biosensor. The fluorescence behavior of the biosensor membrane was recorded at room temperature and λ_ex_ = 320 nm wavelength excitation. Fluorescence intensities of the biosensor membrane steadily decreased with increased sucrose concentrations, with no effect on spectral position and shape. In the present study, glucose, the product of sucrose hydrolysis, not only acted as a quencher, but also as a reducing agent for gold produced in the vicinity of nano gold clusters [39-41]. The same principle was used by Scampicchio et al., where the reaction of glucose (produced by an invertase) with gold salt in alkaline media was used for sucrose sensing [42]. Sensor output was expressed by the change in fluorescence intensity relative to the sucrose concentration (ΔFL/ΔSuc) [39]. The quenching reaction progress was observed for 5 min after sucrose addition for this sensor. However, a typical fast quenching response due to invertase action was observed, within 30 seconds (less than1 min) as shown in Figure 5b. Therefore, the response time for the current sensor was superior to previously reported absorbance-based sucrose sensors [28,43,44]. An additional advantage to note is that these fluorescent biosensor membranes retained invertase activity for one week when stored at 4°C in acetate buffer.

The linear dynamic range for a sucrose standard obtained was 2.25×10^−9^ to 4.25×10^−8^ M, and the limit of detection was 2×10^−9^ M (Figure 5c). The R^2^ value was 0.952 which indicates a strong positive relationship of the calibration. Sucrose concentrations < 2×10^−9^ M were not differentiated from the reference spectrum. Therefore, this was designated as a cut-off value and limit of detection. This threshold was attributed to the limited amount of invertase-nanogold conjugates that can react with sucrose in an onion membrane. The presently designed microplate readout sensor is found to be faster with less sample volume compared to other transducer-based biosensors [28,43-45]. Table 2 summarizes the sensor analysis times, dynamic range and sensitivities of the various sucrose biosensors reported previously.

**Table 2:** **Comparison of the sensor readout time, dynamic range and limit of detection for different types of sucrose biosensors**

**Type of sensor**	**Readout time**	**Dynamic linear range**	**Lower detection limit**	**Reference**
Amperometric Sucrose-fructose biosensor	8 s	1x10^−4^ to 5x10^−3^ M.	2 x10^−6^ M	[46]
Microbial sensor based on *E. coli* strain K-802	Real time	5x10^−5^ to 5x10^−4^ M	5x10^−5^ M	[45]
Microbial sensor based on *B. subtilis* strain VKM B-434	Real time	5x10^−6^ to 5x10^−5^ M	5x10^−6^ M	[45]
Conductometric tri-enzyme biosensor	1–2 min	2x10^−6^ to 5x10^−3^ M	2x10^−6^ M	[43]
Electrochemical tri-enzyme based sensor	1 min	4x10^−6^ to 8x10^−6^ M	4.5x10^−6^ M	[44]
Quenching based on INV-NAuCs-Om sensor	30 s	2.25x10^−9^ to 4.25x10^−8^ M	2x10^−9^ M	[Present biosensor]

### Spiked samples testing

The feasibility of a quenching biosensor for sucrose detection was evaluated by analysis of green tea samples. Measurements with the fluorescence biosensor were further validated against a standard analytical dinitrosalicylic (DNSA) method. Green tea samples spiked with various sucrose concentrations were prepared for testing, while for blank correction for fluorescence spectra, green tea without sucrose was used. Samples were analyzed using the current biosensor at room temperature (after appropriate dilutions). The comparison of testing results between INV-NAuCs-Om biosensor and DNSA analysis of the sucrose-spiked samples are shown (Table 3). These two methods showed a high compliance, with an acceptable error. Satisfactory recoveries ranging from 94 to 108% were obtained, indicating acceptable accuracy of the proposed detection sensor for sucrose in green tea samples.

**Table 3:** **Spike sample testing with a fabricated INV-NGC-Om sensor**

Green tea*	**Spiked concentration (ng mL** ^**−1**^ **)**	**DNSA method (ng mL** ^**−1**^ **)**		**INV-NAuCs-Om Sensor Output (ng mL** ^**−1**^ **)**		
		**Detected concentration**	**% Error**	**Detected concentration**	**% Error**	**Recovery (%)**
Spike A	25	26	4.0	24	−4.0	96.0
Spike B	50	51	2.0	47	−6.0	94.0
Spike C	75	75	0.0	77	2.6	102.67
Spike D	100	108	8.0	108	8.0	108.0
Spike E	125	127	1.6	119	−4.8	95.20
Spike F	150	155	3.3	160	6.6	106.67

*Data represented an average of three independent experiments.

## Conclusion

Our present work is apparently the first to use a fluorometric optical onion membrane-based sensor for detection of sucrose. The sensor was based on formation of invertase-induced nanogold clusters and particles within the membrane. Sucrose was hydrolysed by invertase to glucose, which in turn quenched fluorescence. The microplate-based biosensor yielded comparable results with a traditional method for quantifying sucrose in green tea, providing evidence of reliable sensitivity. Therefore, the proposed fluorescent biosensor has potential as a sensitive one-step measurement of sucrose. Furthermore, after some modifications and future investigations, we expect that this technology can be used to estimate glucose concentrations in various sugar-sweetened beverages and other food products. Likewise, this application can be easily adapted in pharmaceutical research where routine screening of glucose is mandatory.

## Methods

### Materials

Invertase from baker’s yeast (*Saccharomyces cerevisiae*; EC 3.2.1.26) was purchased from Fluka (Milwaukee, WI, USA). Albumin from bovine serum, glutaraldehyde, sucrose, hydrogen tetrachloroaurate, trisodium citrate, gold chloride and glucose were purchased from Sigma-Aldrich (St. Louis, MO, USA). Ultra-pure water filtered by Millipore SAS 67120, Molsheim, France, was used for all experiments. All other chemicals were of the highest purity and used without further purification.

### Invertase assay and immobilization

Large yellow onion bulb (*Allium cepa* L.) with significantly high natural organosulfur compounds which are also flavor precursors of onion were used for the present study. Fully mature onions were purchased from a local vegetable market (Taipei, Taiwan). The onions were cut into halves, bulb scales separated and inner epidermis was stripped from the outer fleshy scales [22]. This thin bulb epidermal cell wall was used as support for invertase immobilization and matrix for nano gold synthesis. The onion membranes (diameter, 6.0 mm) were thoroughly washed and then incubated with glutaraldehyde (0.01%) in acetate buffer (pH 4.5) at 4°C in the dark, for 1 h. Activated membranes were washed gently three times to remove excess glutaraldehyde and treated with a mixture of invertase (500 μL, 220 U mL^−1^) and bovine serum albumin (1 mL, 1 mg mL^−1^) at 4°C for 12 h under gentle stirring. The onion membranes with immobilized invertase were washed twice and tested for enzyme activity using the DNSA method with sucrose (2.5×10^−8^ M) as substrate [26-28]. Invertase-immobilized onion membranes were then stored at 4°C until use. One unit of invertase activity was defined as the amount of enzyme that hydrolyzed 1 μmole of sucrose in 1 min at 30°C in sodium acetate buffer (20 mM, pH 4.5).

### Invertase-mediated nano gold synthesis

Invertase-immobilized onion membranes were immersed in a mixture of 1.0 mL hydrogen tetrachloroaurate (0.5 mM) and 1.0 mL of assay buffer for 24 h at 55°C in a shaking incubator. The transparent, thin onion membranes changed from colorless to a slight yellowish pink color, indicating nanogold synthesis during incubation. Resulting membranes were stored at 4°C in acetate buffer (pH 5.0).

### UV–vis absorbance spectroscopy

UV-Visible spectra analysis was used to confirm reduction of hydrogen tetrachloroaurate (HAuCl_4_) and formation of nanogold on the invertase-bound onion membranes. Biosynthesis of invertase-assisted nanogold clusters and nanoparticles on the Oms were monitored periodically for 72 h. The Om samples were scanned from 300 to 600 nm wavelengths using a dual beam UV-Visible spectrophotometer (1 nm resolution). The UV–vis spectra of the immobilized membranes in assay buffer solution was measured and compared to blank onion membranes.

### Fluorescent imaging

The invertase-immobilized onion membranes (1.0 × 1.0 cm^2^) both with and without nanogold were analyzed under fluorescence imaging using a Leica MZ16F fluorescence stereomicroscope equipped with a DFC 500 camera having GFP filter from Leica Microsystems (Switzerland) Ltd. A magnification range 7.1× to 115×, with a 10× eyepiece, was used to obtain optical images (exposure time of 10.41 s and gain of 80.4%). Images were analyzed with Leica image manager 50, V1.20 software.

### SEM images of the onion membrane

A scanning electron microscope (Model JEOL JSM-6300 F, Tokyo, Japan; 2–5 kV with Auto Fine Coater, JEOL-JFC-1600E Ion Sputtering Device) was used to study modified invertase-onion membranes. During SEM analysis, onion membrane(s) were mounted on stubs and coated with Au/Pd. SEM micrographs of both the invertase immobilized and those that were blank were taken at various magnifications.

### TEM study of onion membrane

The invertase-onion membranes with nanogold clusters were also analyzed by transmission electron microscopy to identify the effects of pH on nanogold synthesis. The INV-NAuCs-Om was cut into circles (~ Φ3 mm) using a razor at room temperature. Membrane pieces were supported on a conventional Φ3 mm Cu mesh with a carbon micro-grid. The TEM observations were performed using a JEOL JEM-3000 F transmission electron microscope (Topcon Co., Ltd., Japan) operated at an accelerating voltage of 300 kV.

### Fluorescence lifetime of INV-NAuCs-Om

Fluorescence lifetime data for light-emitting NAuCs-onion membranes were obtained with an FLS920 combined steady-state lifetime fluorescence spectrometer (Hitachi, Japan). Decay curves were analyzed with a multi-exponential iterative fitting program provided with the instrument. The quantum yield (QY) of INV-NAuCs-Om and INV-Om were determined using l-tryptophan as a criterion (QY = 0.14) at room temperature.

### Sensor fabrication and sucrose measurement

To develop a simple read-out and highly sensitive biosensor system, we used a 96-well fluorescence-compatible microplate as a convenient platform. There are many reports of innovative optical and electrochemical biosensors using a microplate as a reusable component of biosensor [22,29,30]. Black polystyrene FluoroNunc™/LumiNunc™ plates (with minimum back-scattered light and background fluorescence) were used for the measurements. Tecan Infinite® 200 PRO microplate reader with Tecan i-control software was used for microplate analysis. The INV-NAuCs-Om disc (Φ 5 mm) was prepared and adhered on a cover glass disc, without any adhesive, with the hydrophobic side of the onion membrane downward. These modified glass sensor chips were placed at the bottom of each well of the microplate cassette. The INV-NAuCs-Om microplate sensor was calibrated using a standard sucrose solution and fluorescence measurements were recorded. After sensor characterization, sucrose-spiked real samples were also tested. An INV-NAuCs-Om modified microplate was used as a transducer tool to evaluate performance of bioconjugated membranes. For this, 25 μL buffered sucrose solution (2.5×10^−8^ M) was added to the sensing zone and fluorescence was analyzed by exciting the probe at 320 nm. Thereafter, sucrose concentration was increased to 4.25×10^−8^ M, and fluorescence intensity at 348 nm of the INV-NAuCs-Om sensor was measured. Experiments were repeated at least three times, and similar results were obtained. One representative set of data was used for analysis as described.

### Preparation and determination of sucrose in spiked samples

Spike recovery is important to investigate the accuracy of an analytical method. The applicability of the fabricated sensor was evaluated using green tea samples obtained from a local market (Taipei, Taiwan). Tea samples were filtered through a 0.22 μm membrane prior to dilution with assay buffer (acetate buffer, 20 mM, pH 5.0). Spiked tea samples were prepared with 25–150 ng mL^−1^ sucrose concentrations. For testing, 25 μL buffered spiked sample solution was drop-tested on the sensor zone and fluorescence analysis was performed by exciting the INV-NAuCs-Om probe at 320 nm. The quenched fluorescence signal was monitored for all tea samples.

## Additional file

Additional file 1:
**Invertase-nanogold based quenching sucrose sensor.**

## Abbreviations

INV: Invertase
Om: Onion membrane
AuNPs: Gold nanoparticles
NAuCs: Nanogold clusters
DNSA: Dinitrosalisilic acid
TEM: Transmission electron microscopy
SEM: Scanning Electron Microscopy
AFM: Atomic force microscopy
FL: Fluorescence
