# Supplementary material for: Invertase-nanogold clusters decorated plant membranes for fluorescence-based sucrose sensor
Source: J Nanobiotechnology. 2015 Apr 12;13:30. doi: 10.1186/s12951-015-0089-1 (PMC4415262; doi:10.1186/s12951-015-0089-1)
Supplement: Additional file 1: — Invertase-nanogold based quenching sucrose sensor. [file 12951_2015_89_MOESM1_ESM.docx]

**INVERTASE-NANOGOLD CLUSTERS DECORATED PLANT MEMBRANES FOR FLUORESCENCE-BASED SUCROSE SENSOR**

**Dipali Bagal-Kestwal, Rakesh Mohan Kestwal, Been-Huang Chiang***

*Institute of Food Science and Technology, National Taiwan University,*

*No.1, Roosevelt Road, section 4, Taipei, Taiwan, ROC.*

***** Corresponding to:

Tel.:+886233664119; fax:+886223620849.

E-mail address: bhchiang@ntu.edu.tw (B. H. Chiang)

**S1.1. Influence of pH on nanogold clusters synthesis**

It is well established that pH is a factor influencing nucleation and growth of AuNPs [1, 2, 3]. For building plasmonic nanogold sucrose sensors, it is important to consider effects of pH on synthesis and stability of sensing nanogold clusters. Normalized absorption spectra of the INV-NAuCs-Om for which the gold nanoparticles synthesized at pH ranged from 3.0 to 11 (Figure S1). As previously reported by researchers, the aggregation of nanogold broadened the absorption spectrum and shifted the SPR peak [4, 5]. Changes in pH directly affect interactions among invertase, onion membrane and nanogold in terms of Van der Waals forces, repulsive electrostatic forces and other small weak binding forces. Elliptical tubular nano-sized particles were observed in acidic pH (range, 3.0 to 6.0; Figure S2 (a-d)). Due to invertase, the smaller gold nuclei effectively aggregated into sub-micrometer size with clusters. At pH 5.0, there was another plasmon minor peak (λ_max_ =538 nm), confirming the existence of free nanogold particles (various sizes), which were visualized by TEM (Figure S2), including large structures due to aggregation of nanogold particles.

Distinct peaks at UV region due to INV-bound nanogold clusters occurred at pH ranged from 4 to 6 (Figure S2 (a-d)). However, from pH 7.0 onwards, the minor peak (λ_max_ from 500 to 650 nm) become more distinct, due to growth of scattered nanoparticles, indicating weaker nano-protein conjugation leading to formation of dispersed nanoparticles (Figure S1 (e-i)). These results were confirmed by TEM analysis, with typical bone-shaped elongated nanoparticles observed (Figure S2 (e-i)). These microscopic observations were consistent with UV-Vis spectroscopic studies.^8^ Nanoparticles of various sizes have their own characteristic surface plasmon resonance peaks, manifest in differences in UV-Visible spectra, which in turn can affect particle size. At pH 8, polydispersed nanogold particles with an average diameter of 11 nm ± 20 were observed, with an intense UV-visible peak at 547 nm. Furthermore, gold particles isolated from the onion membrane were more uniform in both shape and size under alkaline conditions (pH 9.0 to 11; Figure S1 (g-i)). However, when synthesis was done at high pH (>7.0), the decrease in absorbance peak with large background noise was observed in UV region. Under these extreme pH conditions, invertase may have lost its active biological scaffold and resulted in non-fluorescent gold nanostructures. Nanoparticles with major and minor SPR peaks around 550 nm indicated that various sizes of nanoparticles were formed at pH 10. At pH 11, tiny spherical nanoparticles (range of 1-10 nm ± 18) were synthesized. Furthermore, the absorption band for major peak was shifted to 547 nm, indicating the alkaline chemical reduction of gold with increase particle size at this pH. According to Popescu et al. an extracellular secretion of enzymes facilitates getting large quantities of nanoparticles of size 100 – 200 nm in a relatively pure state, free from other cellular proteins [6]. This rational biosynthesis strategy may apply to the present situation. However, as pH increases, not only invertase but also other proteins from inner onion epidermal membrane may also lose both their three-dimensional conformation and their biological catalytic activities.


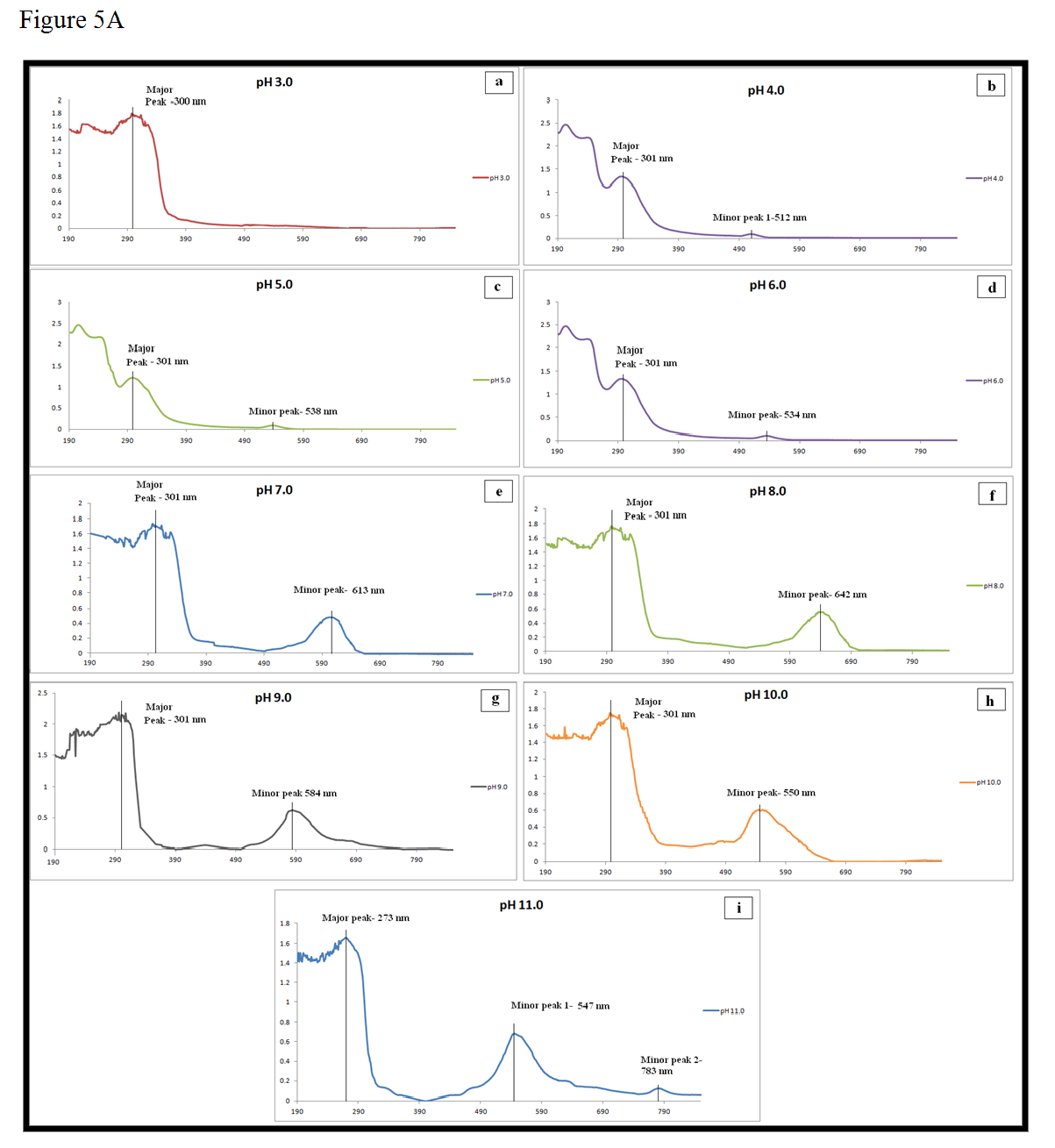


**Figure S1. UV-visible absorbance spectra of nanogold structure synthesis as a function of pH.**

**
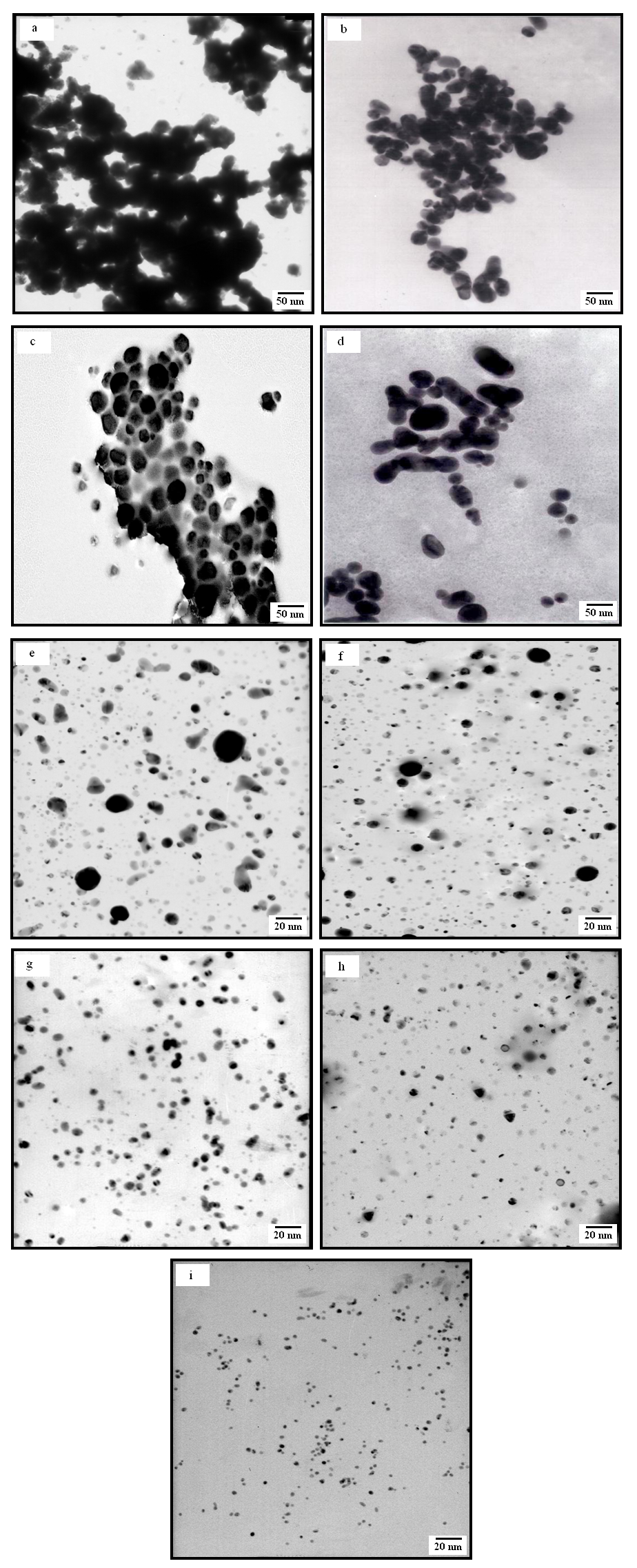
**

**Figure S2. TEM micrographs of nanogold when prepared at various pH: a) pH 3.0, b) pH 4.0, c) pH 5.0, d) pH 6.0, e) pH 7.0, f) pH 8.0, g) pH 9.0, h) pH 10.0, and i) pH 11.0, respectively.**

**
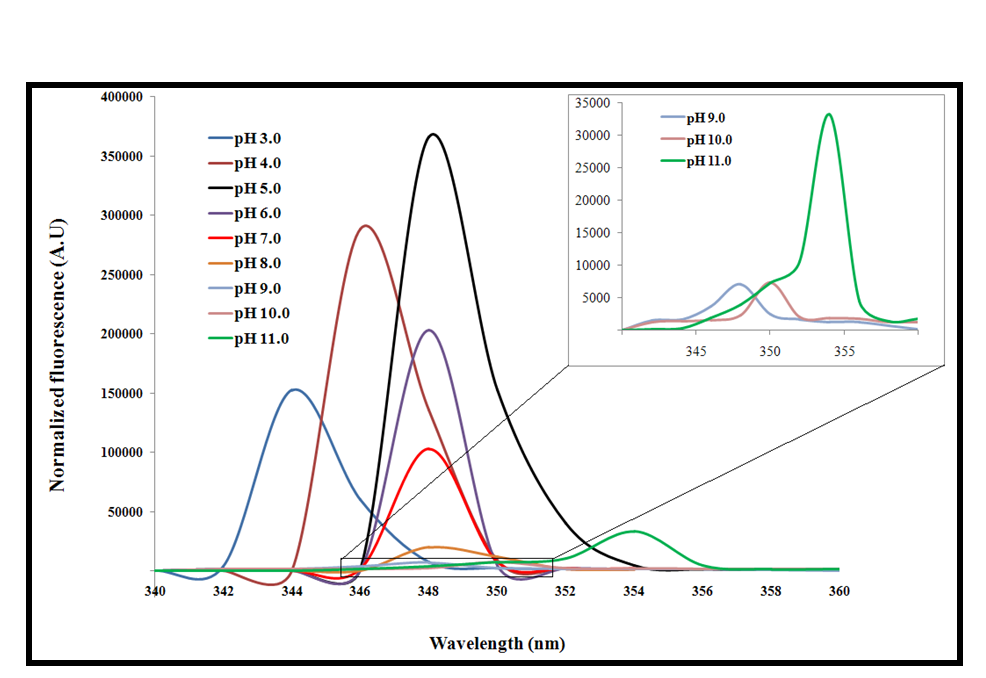
**

**Figure S3. pH-dependent intrinsic fluorescence of nanogold clusters in aqueous solutions with various pH values.**

**S1.2. Effect of invertase concentration on nanogold clusters synthesis**

All spectra had the same plasmonic band with a maximum at 346 nm when excited at 320 nm, which was intensified as the concentration of invertase increased (see Figure S4, Inset). However, when the invertase exceeded 330 Units, fluorescence intensity no longer increased, perhaps due to the masking of enzyme at high concentrations, as well as nanoparticle aggregation.


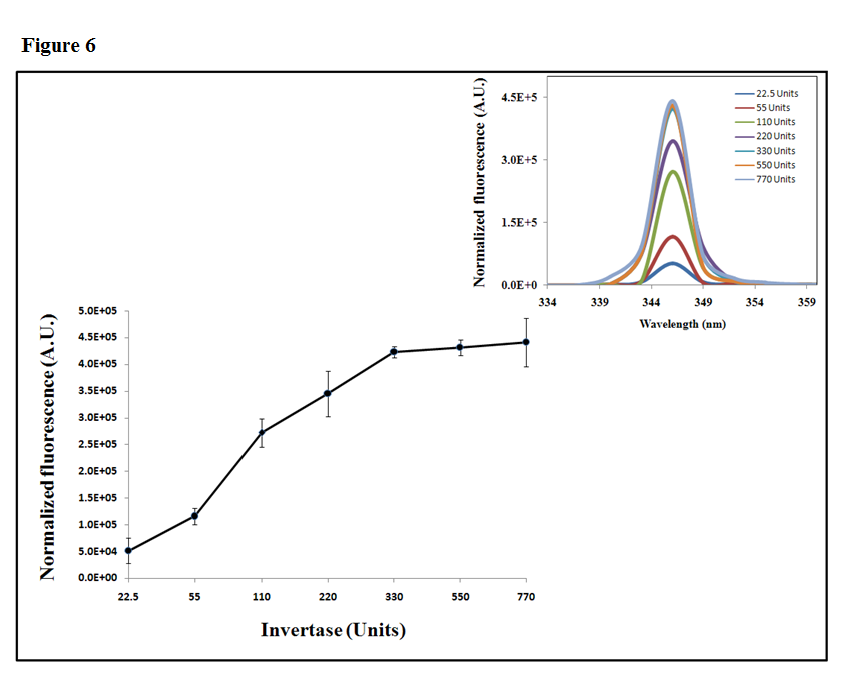


**Figure S4. Effect of invertase on nanoparticle formation in onion membrane. Inset: Fluorescence emission spectra of INV-NAuCs-Om *versus* invertase when excited at 320 nm.**

**References**

1. Turkevich J, Stevenson PC, Hillier J: **Formation of colloidal gold**. *J Phys Chem* 1953, **57**: 670-673.
2. Park SE, Park MY, Han PK, Lee SW: **The effect of pH-adjusted gold colloids on the formation of gold particles over APTMS-coated silica cores**. *Bull Kor Chem Soc* 2006, **27**:1341-1345.
3. Wang S., Qian K., Bi X., Huang W: **Influence of speciation of aqueous HAuCl_4_ on the synthesis, structure, and property of Au colloids**. *J Phys Chem* C 2009, **113**: 6505-6510.
4. D. Roll, J. Malicka, I. Gryczynski, Z. Gryczynski, J.R. Lakowicz, Metallic colloid wavelength-ratiometric scattering sensors. Anal. Chem. 75 (2003) 3108-3113.
5. K. Aslan, F.R. Lakowicz, C.D. Geddes, Nanogold-plasmon-resonance-based glucose sensing Anal. Biochem. 330 (2004) 145-155.
6. M. Popescu, A. Velea, A. Lőrinczi, Biogenic production of nanoparticles. Dig. J. Nanomater. Bios. 5 (2010**)** 1035-1040.
